# Fluoroscopy-assisted posterior percutaneous reduction for the management of unilateral cervical facet dislocations after unsuccessful closed reduction: A case report

**DOI:** 10.1016/j.ijscr.2019.04.038

**Published:** 2019-05-03

**Authors:** Takaki Shimizu, Katsuhito Yoshioka, Hideki Murakami, Satoru Demura, Satoshi Kato, Noriaki Yokogawa, Norihiro Oku, Ryo Kitagawa, Hiroyuki Tsuchiya

**Affiliations:** Department of Orthopaedic Surgery, Graduate School of Medical Sciences, Kanazawa University, 13-1 Takara-machi, Kanazawa, 920-8641, Japan

**Keywords:** ACDF, Anterior cervical discectomy and fusion, Anterior cervical surgery, Cervical facet dislocation, Percutaneous reduction

## Abstract

**Introduction:**

In some cases of cervical facet dislocations, open reduction becomes imperative when closed reduction fails. In these cases, posterior open reduction with subsequent posterior fixation has been favored in previous reports as reduction using the posterior approach is less challenging than that using the anterior approach. However, it invades the posterior cervical muscles, is associated with a high risk of postoperative axial neck pain, and is less likely to restore cervical lordosis than anterior surgery. In this report, we describe a novel reduction technique, posterior percutaneous reduction, which can address this dilemma.

**Presentation of case:**

An attempt to perform closed reduction in a 19-year-old adolescent with a unilateral facet dislocation at the C4-C5 level was unsuccessful. To preserve the posterior cervical muscles and obtain good cervical alignment, we opted for posterior percutaneous reduction and subsequent anterior cervical discectomy and fusion instead of posterior open reduction and fixation. An elevator was inserted into the locked facet percutaneously with fluoroscopic assistance, and reduction was achieved by lever action. Seven days after the percutaneous reduction, anterior cervical discectomy and iliac bone grafting with plate fixation were performed. There were no complications or neurological deficits postoperatively.

**Discussion:**

This report describes the case of a patient who underwent anterior cervical discectomy and fusion after posterior percutaneous reduction with preservation of the posterior cervical muscles for unilateral facet dislocation when closed reduction was unsuccessful.

**conclusion:**

Posterior percutaneous reduction could be a useful option for the management of cervical facet dislocations.

## Highlights

• Open reduction of cervical facet dislocation is needed when closed reduction fails.
• Anterior cervical discectomy and fusion after posterior percutaneous reduction was performed.
• Posterior percutaneous reduction can be useful for cervical facet dislocations.

## 1 Introduction

For patients with cervical facet dislocations, closed reduction may play a role in the initial treatment. Anterior cervical discectomy and fusion (ACDF) is usually performed after successful closed reduction of cervical facet dislocations without posterior bone compressions, such as that on movement of a laminar fragment into the canal or on significant bone disruption. However, in the event of failed closed reduction, open reduction is required. In these cases, posterior open reduction and subsequent posterior fixation are performed, as discussed in previous reports. [1,2] Reduction via the posterior approach is less challenging than that via the anterior approach. However, it invades the posterior cervical muscles and is associated with a high risk of postoperative axial neck pain [3]. In addition, posterior approaches are less likely to restore cervical lordosis than are anterior approaches [4]. The absence of a normal cervical alignment may also negatively influence the long-term outcomes. Herein, we suggest a novel reduction technique, the posterior percutaneous reduction, which can address this dilemma. In this report, we describe the case of a patient who underwent ACDF after posterior percutaneous reduction for unilateral facet dislocation, for which closed reduction was unsuccessful. This report fulfills the SCARE criteria [5].

## 2 Presentation of case

**Fig. 1:**
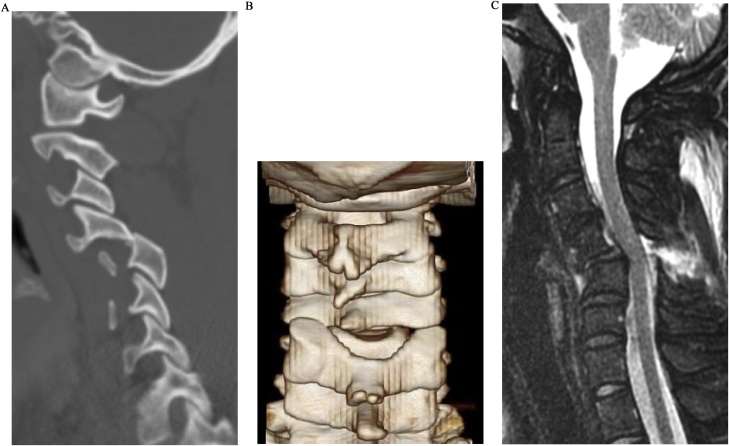
**a)** Computed tomography (CT) images showing left facet dislocation at C4-C5. **b)** Three-dimensional CT reconstruction of the cervical spines, showing a dilated interlaminar space. **c)** Magnetic resonance images showing spinal cord compression at C4-C5 on T2 STIR-weighted sequence with no obvious disc herniation.

**Fig. 2:**
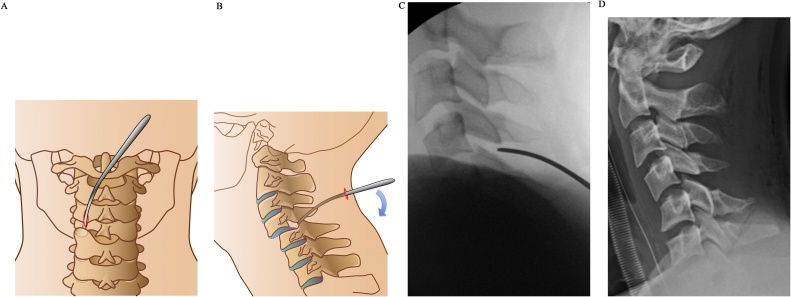
**a, b)** Schematic illustration of the posterior percutaneous reduction with an elevator. **c)** Fluoroscopic image after percutaneous reduction with an elevator. **d)** Radiograph after reduction showing 18°-kyphosis and a dilated interspinous space at C4-C5.

**Fig. 3:**
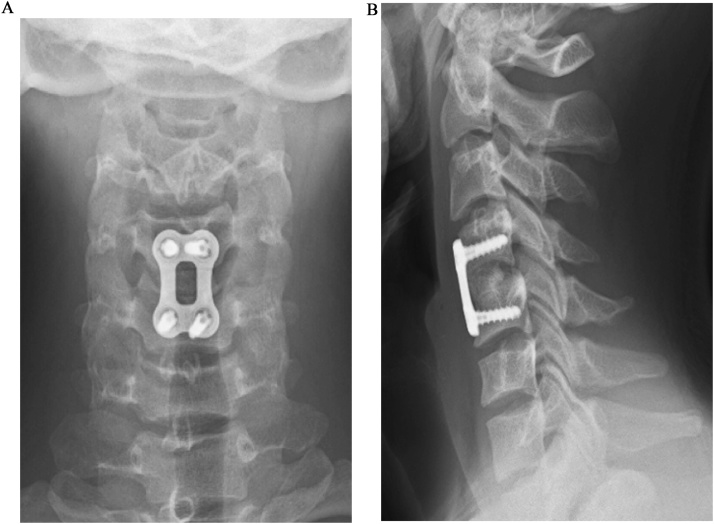
Anteroposterior (**a**) and lateral (**b**) radiographs 3 months after ACDF showing complete bony union and 4°-lordosis.

A 19-year-old adolescent was involved in a motor vehicle accident that resulted in a unilateral facet dislocation at the C4-C5 level. Although magnetic resonance imaging showed spinal cord compression at the injured segment, the patient had no neurological deficits and presented with score E on the American Spinal Injury Association impairment scale (Fig. 1). An attempt to perform closed reduction with halo crown traction under mild sedation immediately after transfer to the hospital was unsuccessful. To preserve the posterior cervical muscles and obtain good cervical alignment, posterior percutaneous reduction and subsequent ACDF were selected, instead of posterior open reduction and fixation. Six hours after injury, the patient was placed in the prone position using a Mayfield head holder under general anesthesia. An elevator was inserted into the locked facet percutaneously through a small incision above the facet with fluoroscopic assistance, and reduction was achieved by lever action without complications (Fig. 2). Seven days after the percutaneous reduction, anterior cervical discectomy and iliac bone grafting with plate fixation were performed. The postoperative cervical alignment was optimal, and complete bony union was achieved (Fig. 3). There were no complications or neurological deficits postoperatively.

## 3 Discussion

Early reduction is recommended for acute management of cervical facet dislocation to decompress the spinal cord and improve neurological outcomes [4,[6], [7], [8], [9]]. Closed reduction is a common initial treatment because up to 80% of dislocations can be reduced with external traction [10]. Cervical facet joint dislocation is generally managed surgically after reduction. The Subaxial Injury Classification System and Severity Score [11] recommends that unilateral or bilateral facet dislocation be managed surgically, even in the absence of spinal cord injury [7,12]. Conservative management is associated with a high incidence of recurrent instability and long-term pain and disability [2,13,14].

Although there is insufficient evidence of superiority between the anterior and posterior approaches for the management of cervical facet dislocation, [15] Kwon et al. reported that patients treated with ACDF for unilateral facet fracture dislocation had lesser postoperative pain, a lower rate of wound infection, a higher rate of radiological bone union, and better cervical alignment than did patients treated using a posterior approach [4]. Anterior approaches have the advantages of the supine position and direct anterior decompression of neural elements, such as a disk herniation, or an anteriorly located bone fragment. In addition, anterior approaches are less invasive to the cervical muscles and are not associated with the risk of postoperative axial neck pain, unlike the posterior approach [3].

As for the reduction technique, some authors suggest performing open reduction and stabilization using an anterior approach if closed reduction fails. Many different surgical techniques have been proposed to reduce facet dislocations using an anterior approach. However, these techniques do not involve direct reduction, unlike the posterior approaches. Some cases may require additional posterior open surgery to reduce the dislocations if anterior reduction fails. [9,16] These difficulties in reduction and additional invasiveness are the main drawbacks of using an anterior approach. Therefore, in cases involving closed reduction failure, posterior cervical surgery cannot be precluded, because it provides direct reduction although there are several disadvantages with posterior approaches.

In the present case, reduction was successfully achieved via a posterior percutaneous approach, with the advantages of both direct reduction and cervical muscle preservation. Subsequent ACDF resulted in good cervical alignment without complications. Percutaneous reduction can be achieved early with less invasive decompression of the neural tissue, without the need for special devices or skills in spine surgery. While performing this technique, careful attention must be paid to avoid inserting the elevator into the spinal canal, as the interlaminar space is dilated because of facet dislocation and posterior ligamentous rupture (Figs. 1b, 2 a). This treatment technique, which reduces the dislocation using the posterior approach and fixes it using the anterior approach, utilizes the advantages of both approaches and can be applied in most cases without posterior compression or comminuted fractures.

## 4 Conclusion

Thus, we achieved reduction for unilateral cervical facet dislocation after unsuccessful closed reduction using a posterior percutaneous approach with preservation of the posterior cervical muscles and subsequent ACDF, resulting in a good clinical course. This novel reduction technique, which allows early and less invasive decompression of the neural tissue without special devices, could be a useful option for the management of cervical facet dislocations. Further investigation is necessary to establish the efficacy and safety of this technique.

## Conflicts of interest

The authors have no conflicts of interest to declare.

## Sources of funding

None.

## Ethical approval

Our institution’s ethics committee approved the study and the reference number is 2015−075 (1893).

## Consent

The patient provided informed consent for the publication of this report.

## Registration of research studies

This study was registered as a case report on the www.researchregistry.com website with UIN 4598.

## Guarantor

Takaki Shimizu, MD

## Provenance and peer review

Not commissioned, externally peer-reviewed.

## CRediT authorship contribution statement

**Takaki Shimizu:** Data curation, Writing - original draft. **Katsuhito Yoshioka:** Methodology, Writing - review & editing. **Hideki Murakami:** Conceptualization. **Satoru Demura:** Writing - review & editing. **Satoshi Kato:** Validation. **Noriaki Yokogawa:** Data curation. **Norihiro Oku:** Data curation. **Ryo Kitagawa:** Data curation. **Hiroyuki Tsuchiya:** Supervision.
